# Differential Biodistribution of Adenoviral-Vectored Vaccine Following Intranasal and Endotracheal Deliveries Leads to Different Immune Outcomes

**DOI:** 10.3389/fimmu.2022.860399

**Published:** 2022-06-10

**Authors:** Vidthiya Jeyanathan, Sam Afkhami, Michael R. D’Agostino, Anna Zganiacz, Xueya Feng, Matthew S. Miller, Mangalakumari Jeyanathan, Michael R. Thompson, Zhou Xing

**Affiliations:** ^1^ McMaster Immunology Research Centre, M. G. DeGroote Institute for Infectious Disease Research, Hamilton, ON, Canada; ^2^ Department of Medicine, McMaster University, Hamilton, ON, Canada; ^3^ Department of Biochemistry & Biomedical Sciences, McMaster University, Hamilton, ON, Canada; ^4^ Department of Chemical Engineering, McMaster University, Hamilton, ON, Canada

**Keywords:** respiratory mucosal immunization, intranasal, endotracheal, biodistribution, Adenovirus-vectored vaccine, Tuberculosis, mucosal immunity, T cells

## Abstract

Infectious diseases of the respiratory tract are one of the top causes of global morbidity and mortality with lower respiratory tract infections being the fourth leading cause of death. The respiratory mucosal (RM) route of vaccine delivery represents a promising strategy against respiratory infections. Although both intranasal and inhaled aerosol methods have been established for human application, there is a considerable knowledge gap in the relationship of vaccine biodistribution to immune efficacy in the lung. Here, by using a murine model and an adenovirus-vectored model vaccine, we have compared the intranasal and endotracheal delivery methods in their biodistribution, immunogenicity and protective efficacy. We find that compared to intranasal delivery, the deepened and widened biodistribution in the lung following endotracheal delivery is associated with much improved vaccine-mediated immunogenicity and protection against the target pathogen. Our findings thus support further development of inhaled aerosol delivery of vaccines over intranasal delivery for human application.

## Introduction

Infectious diseases of the respiratory tract are one of the top causes of global morbidity and mortality, with lower respiratory tract infections being the fourth leading cause of death worldwide in 2019 (1). In fact, the COVID-19 pandemic is a sobering example of the extent of a threat that a respiratory mucosal infection can cause to humankind (2). Vaccination is the most cost-effective public health measure to prevent or control respiratory infectious diseases. However, the vast majority of current vaccines including anti-tuberculosis (TB) BCG in human immunization program are administered *via* a parenteral route and thus, induce only limited respiratory mucosal immunity against respiratory pathogens such as *Mycobacterium tuberculosis* (*M.tb*) and influenza (3–5). This reality calls for continuing effectors to develop respiratory mucosal vaccine strategies (3, 6). In this regard, on top of injectable flu shots, an intranasally administered live-attenuated influenza vaccine has been introduced to human immunization program as the first respiratory mucosal-deliverable vaccine in humans. Unfortunately, while this nasal flu vaccine shows high efficacy in young children, it is much less effective in adults than injectable flu shots (5, 7). This explains the reason why the injectable flu vaccine remains the top choice for seasonal flu vaccination in general populations. These facts question the suitability of intranasal vaccine delivery as a general respiratory mucosal vaccine strategy for human application. Recently, as an alternative respiratory mucosal vaccine strategy, inhaled aerosol delivery method has been developed and explored to deliver measles vaccine (8), viral-vectored TB vaccines (9, 10) and a viral-vectored COVID-19 vaccine (11) in human trials. Of importance, when parenteral intradermal or intramuscular route of vaccination was compared side-by-side with inhaled aerosol vaccination, it was found that only inhaled aerosol, but not parenteral, vaccination induced significant respiratory mucosal immunity (9, 10). Since inhaled aerosol technology bypasses the nasal passage and delivers the vaccine droplets of 2-5 µm deep into human respiratory tract (airways) (10), these clinical observations together appear to suggest that the biodistribution or the depth of respiratory vaccine delivery plays a critical role in induction of respiratory mucosal immunity. However, to firmly prove this proposition requires experimental investigation in preclinical animal models since it is very difficult to directly test it in humans.

Unfortunately, to date there has been a paucity in experimental studies to compare intranasal delivery with intratracheal/endotracheal deep-airway delivery in vaccine biodistribution, vaccine-specific mucosal immune responses, and protective efficacy. Although there are experimental studies that suggest intratracheal delivery of non-vaccine biologic agents including LPS and microbes to lead to deeper/wider biodistribution and/or manifestation of tissue inflammation, over the intranasal delivery method (12–14), other studies report the opposite observations (15, 16). To our knowledge, there are only two experimental studies where intranasal and intratracheal vaccine delivery methods were compared but these studies did not assess vaccine biodistribution and/or both mucosal T cell immunity and protective efficacy (17, 18). Therefore, there is a need to experimentally address the relationship of differential biodistribution of vaccine delivered by intranasal and intratracheal/endotracheal methods to vaccine-specific mucosal immune responses and protective efficacy. Our enhanced knowledge in this regard will help inform whether going forward, we should focus on developing inhaled aerosol deep-airway vaccine strategies over the intranasal delivery method for human application.

In the current study, using an adenovirus-vectored TB vaccine (AdHu5Ag85A) as a model vaccine, we have evaluated the biodistribution, vaccine immunogenicity and immune protective potency following a single-dose intranasal or endotracheal delivery in a murine model. We find that endotracheal delivery is superior to internasal delivery, which leads to deep lung biodistribution of vaccine, and enhanced vaccine-specific T cell responses and protective efficacy against pulmonary *M.tb* challenge. Our findings thus provide preclinical evidence to support the consideration of the deep-airway delivery method such as inhaled aerosol over the intranasal delivery for human application.

## Materials and Methods

### Mice

Female BALB/c or C57BL/6 mice aged 6 to 8 weeks were sourced from Charles River Laboratories and housed in the Central Animal Facility at McMaster University. All *in vivo* work was done in compliance with guidelines from the Animal Research and Ethics Board at McMaster University and under approved animal utilization protocol (AUP #210822).

### Pulmonary Delivery Methods and *in Vivo* Visualization of Deposition Sites

**Figure 1:**
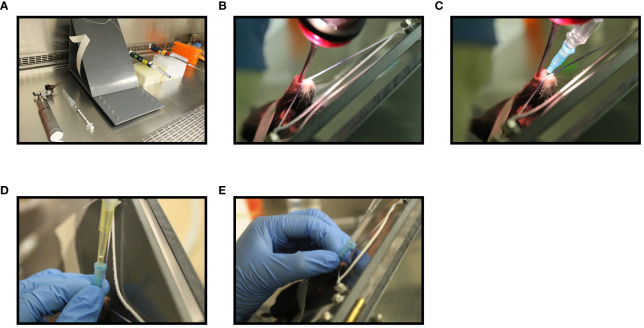
Step-wise illustration of endotracheal delivery method (I.T). Endotracheal intubation was carried out in a C57BL/6 mouse anesthetized with 2% isoflurane and oxygen at a flow rate of 2 liters/min using an illuminated otoscope, a 22G blunt-tip intravenous catheter, a 45^0^ C-angled intubation stand, a P200 pipette with P200 tip and a P200 pipette with extended length gel loading tip **(A)**. Unconscious mouse breathing at a respiration rate of 30 breaths per min was placed by hooking its upper incisor over a string attached to the intubation board. An otoscope was placed into the mouth and the vocal cord was visualized **(B)**. A 22G blunt-tip intravenous catheter attached to a syringe was then inserted into the trachea **(C)**. The syringe was then replaced with a P200 pipette attached to a p200 tip containing water and the movement of water in the tip during breathing was confirmed to affirm the insertion of catheter into the trachea **(D)**. Next, P200 pipette attached to an extended length gel-loading tip loaded with 50 μL AdHu5Luc or vaccine was inserted into the catheter and allow to inhale by the mouse **(E)**.

The impact of different pulmonary delivery methods on the deposition site of a vaccine in the lung was measured using a replication deficient adenovirus expressing luciferase (AdHu5Luc) as a distribution marker. Intranasal inoculation was performed by instilling 5x10^7^ PFU of AdHu5Luc in a total volume of 25 µL of phosphate-buffered saline solution (PBS) into the nostrils of C57BL/6 mice (12.5 µL in each nostril), as described previously (19–21). Endotracheal intubation was carried out using an intubation board set up at 45^°C^, an otoscope, and a 22G blunt-tip intravenous catheter, as described previously (**Figure 1A**) (22). Briefly, after anesthetizing, mice were hung by their teeth on a string attached to the intubation board allowing for easy visualization of the opening of the trachea with the otoscope (**Figure 1B**). Once the opening of the trachea was located, the catheter attached to a syringe was inserted into the trachea (**Figure 1C**). The syringe was then removed and replaced with a P200 pipette containing 100 µL water with a gap of air to confirm correct insertion into trachea (**Figure 1D**). The P200 pipette was then removed and replaced with an extended gel length pipette containing 50 μL of 5x10^7^ PFU AdHu5Luc and was allowed to be inhaled by the mouse (**Figure 1E**). Deposition of AdHu5Luc within the respiratory tract was visualized eight hours post-delivery using an *In Vivo* Imaging System (Caliper Life Sciences). For this, 15 mg/mL of D-luciferin (Caliper Life Sciences) in 25 μL was delivered intranasally as described above and fluorescence signal was visualized within 5 minutes (23). Semi-quantification of strength of fluorescence within the respiratory tract was performed using the ImageJ (Easter Greenbush, NY) image processing program.

### Immunization Using Different Pulmonary Delivery Methods

To assess the consequence of different pulmonary delivery methods on the effectiveness of a vaccine, BALB/c mice were immunized with a well characterized adenoviral-vectored tuberculosis vaccine, AdHu5Ag85A, at a dose of 5x10^7^ PFU/mouse intranasally or by intubation as described above (21, 24, 25). Immunogenicity and protective efficacy were assessed four weeks post-immunization.

### Bronchoalveolar Lavage and Lung Mononuclear Cell Isolation

Mice were euthanized by exsanguination. Cells in bronchoalveolar lavage and lung tissue were isolated as previously described (19–21, 26). Briefly, following exhaustive bronchoalveolar lavage (BAL), left and right lungs were collected separately and cut into small pieces and digested with collagenase type 1 (ThermoFisher Scientific Waltham, MA) at 37°C in an agitating incubator for one hour. A single-cell suspension was obtained by crushing the digested tissue through a 100 μm basket filter (BD Biosciences, San Jose, CA) and lysing the red blood cells using ACK lysis buffer. BAL was centrifuged at 6000 rpm for 3 minutes to pellet the cells. Isolated cells from BAL and the lung were resuspended in complete RPMI 1640 medium (RPMI 1640 supplemented with 10% FBS, 1% L-glutamine, and 1% penicillin/streptomycin).

### Tetramer and Intracellular Cytokine Staining and Flow Cytometry

BAL and lung cells were plated at 2x10^6^ cells/mL and 20x10^6^ cells/mL, respectively, and stimulated with an Ag85a CD8 T cell-specific peptide (MPVGGQSSF) or Ag85a CD4 T cell-specific peptide (LTSELPGWLQANRHVKPTGS) at a concentration of 1 µg/well in the presence of Golgi plug (5 mg/mL brefeldin A; BD Pharmingen) for six hours in a 37°C CO_2_ incubator. For tetramer immunostaining, a tetramer for the immunodominant CD8 T cell peptide (MPVGGQSSF) of Ag85A bound to the BALB/c major histocompatibility complex class I allele (H-2L^d^ conjugated to PE fluorochrome) (NIH Tetramer Core, Atlanta, GA) was used (20). Tetramer stained and stimulated cells were then stained with T cell surface antibodies, followed by fixation/permeabilization by using Fixation/Permeabilization Solution Kit (BD Biosciences, San Jose, CA) according to the manufacturer’s instructions. Cells were then stained with anti-IFN-γ-APC mAb in Perm/Wash buffer (BD Biosciences, San Jose, CA) for 30 min on ice. The monoclonal antibodies used for T cell surface markers were anti-CD3-V450, anti-CD4-APC-Cy7 and CD8-PE-Cy7. All mAbs and reagents were purchased from BD Biosciences (San Jose, CA). Immuno-stained cells were processed according to BD Biosciences instructions for flow cytometry and run on a BD LSR II flow cytometer. Data was analyzed using FlowJo (version 10.1; Tree Star, Ashland, OR).

### *M. Tuberculosis* Preparation and Pulmonary Infection 

*Mtb* H_37_Rv bacilli (H_37_RV; ATCC 27,294) were grown in supplemented Middlebrook 7H9 broth for 14 days as described previously and stored at -70°C (20, 27). Before infection, bacilli were washed twice with PBS containing 0.05% Tween-80 and were subsequently passed 10 times through a 27-gauge needle to dislodge any clumps before *in vivo* usage. Pulmonary infection with the *Mtb* H_37_Rv strain was performed as previously described (20, 27). Briefly, anesthetized mice were intranasally infected with 1x10^4^ CFU of *Mtb* H_37_Rv in 25 μL of PBS. Dosage for infection was verified by plating 10-fold serial dilution on Middlebrook 7H10 agar plate containing Middlebrook oleic acid-albumin-dextrose-catalase (OADC) (Invitrogen Life Technologies, Carlsbad, CA). *Mtb* H_37_Rv burden in the lung was assessed four weeks post-infection by plating serial dilution of lung homogenates in triplicates onto Middlebrook 7H10 agar plates and incubated at 37°C for 21-28 days before enumeration.

### Histological Analysis, Microscopy and Scoring

To assess the impact of different pulmonary delivery methods of vaccine on the lung histopathological changes after pulmonary *M.tb* infection, lung lobes were fixed in paraformaldehyde and subjected to hematoxylin and eosin (H&E) staining. Sections were then scored independently by two researchers blinded for the treatment groups. A scale from 1-10 was used to score the presence of granuloma, pneumonitis, and perivascular and peribronchial infiltration. Images of representative micrographs were taken on a Zeiss Axio Imager 2 Research Microscope using AxioVision digital imaging software (Carl Zeiss Microscopy GmbH, Germany).

### Statistical Analysis

A two-tailed Student t test was performed for pairwise comparisons. One-way ANOVA followed by a Tukey test was performed to compare more than two groups. All analyses were performed on GraphPad Prism (Version 6, GraphPad Software, La Jolla, CA). A p value of <0.05 was considered significant.

## Results

### Heightened Biodistribution in the Lung Following Endotracheal Inoculation Compared to Intranasal Inoculation

**Figure 2:**
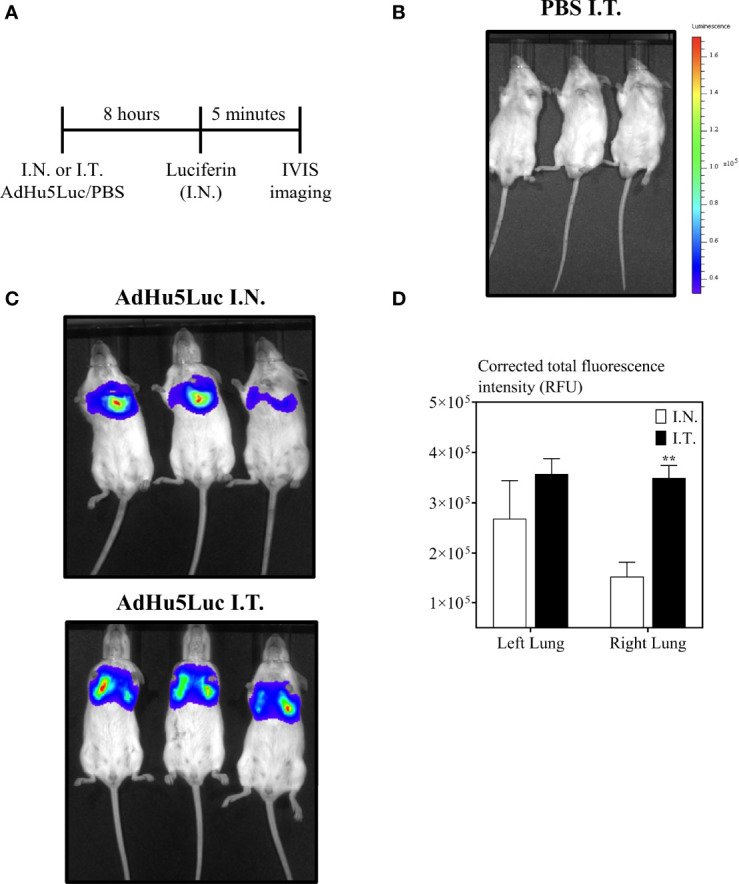
Biodistribution of vaccine surrogate in the lung following intranasal or endotracheal inoculation. **(A)** Experimental schema. Mice were inoculated intranasally (I.N.) or endotracheally (I.T) with adenovirus-vector expressing luciferase (AdHu5Luc) or PBS. Biodistribution was visualized as a factor of light emission upon intranasal administration of luciferin. Images were obtained using an IVIS Spectrum and presented as pseudocolour images of bioluminescence in PBS **(B)** or AdHu5Luc inoculated animals **(C)**. Red represents the most intense areas of biodistribution while the blue corresponds to the weakest areas of biodistribution. Mice were imaged with an integration time of 30 sec. Three mice per treatment group is shown. **(D)** Bar graph shows corrected total fluorescence intensity measured in relative fluorescence units (RFU) and quantified using ImageJ in either right or left lung. Data is from 3 mice/group and RFU are presented as mean ± SEM. **p < 0.01.

Intranasal instillation of 25 μL is the most widely used inoculation method for evaluation of respiratory mucosal delivered novel viral-vectored TB vaccines in mice (28, 29). To begin evaluating the impact of different respiratory route of inoculation on vaccine potency, we first studied the biodistribution of AdHu5Luc, a replication-deficient adenoviral vector expressing luciferase, in the lung as a marker of distribution. To this end, mice were inoculated with an identical dose of 5x10^7^ PFU AdHu5Luc *via* the conventional intranasal (I.N.) delivery or an endotracheal (I.T.) method (Fig.1A-E) and luciferase activity was assessed following administration of luciferin to the lung using IVIS imaging analysis. Luciferase expression was determined at eight hours post inoculation as a measure of corrected total area of fluorescence in left and right lung separately (**Figure 2A**). To define and correct autofluorescence background, PBS treated mice that received luciferin were included. Background signals were not evident in such control mice (**Figure 2B**). Overall, endotracheal delivery led to greater distribution of AdHu5Luc within the lung than intranasal delivery as indicated by much broader fluorescence intensity in both left and right lung lobes (**Figure 2C**). Of note, there was a lack of fluorescence intensity in the right lung of intranasal-inoculated animals. Indeed, upon analysis of the corrected total area of fluorescence intensity (total RFU (mean plus/minus SE) and p value), intranasal inoculation resulted in unequal biodistribution, more being deposited to the left than right lung and was variable between animals (**Figure 2D**). In contrast, biodistribution was comparable between right and left lungs following endotracheal delivery, with significantly higher deposition in the right lung than by intranasal inoculation (**Figure 2D**). These data indicate that different respiratory mucosal delivery methods result in differential biodistribution of adenoviral gene transfer vector within the lung.

### Improved Vaccine-Induced Immunogenicity in the Lung Following Endotracheal Immunization Compared to Intranasal Immunization

**Figure 3:**
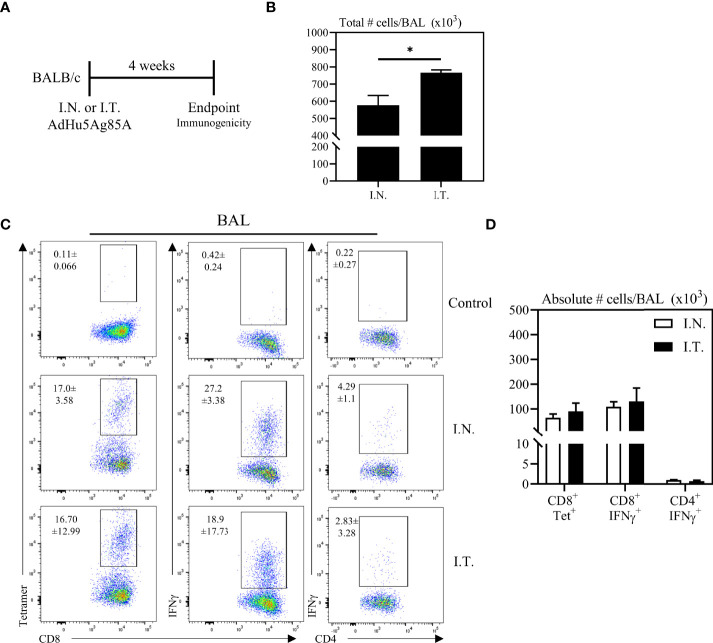
Vaccine-specific T cell responses in the airways following endotracheal immunization compared to intranasal immunization. Experimental schema **(A)**. Mice immunized intranasally (I.N.) or endotracheally (I.T.) with AdHu5Ag85A were scarified 4 weeks post-immunization and mononuclear cells from airways were examined for vaccine-specific responses. **(B)** Bar graphs comparing total number of mononuclear cells in bronchoalveolar lavage (BAL) fluid. **(C)** Representative flow cytometric dotplots showing frequencies of Ag85A-specific CD8 T cells (CD8+tet+) determined by tetramer staining, and frequencies of IFNγ+ CD8+ and CD4+ T cells determined by intracellular cytokine staining of cells stimulated with Ag85A CD8 or CD4 T-cell specific peptides in BAL. Top row dotplots (Control) show the defining gates for tetramer population gated out of total CD8 T cells from unimmunized animal and the gates for CD8+IFNγ+ and CD4+IFNγ+ T cells out of total unstimulated CD8 and CD4 T cells from BAL of immunized mice. Numerical indicated in the dotplots represent the mean frequency of parent (CD4 or CD8 T cells) ± SEM. **(D)** Bar graphs comparing absolute number of CD8+tet+, CD8+IFNγ+, and CD4+IFNγ+ T cells in BAL of intranasal- and endotracheal-immunized mice. Absolute numbers of CD8+tetramer+, CD8+ IFN-γ+ and CD4+ IFN-γ+ shown in bar graphs were calculated based on frequency of CD3+live cells gated out of total events to exclude all non-immune cells. Data is from 3 mice/group, representative of two independent experiments and presented as mean ± SEM.*p < 0.05.

Having demonstrated the broadened biodistribution of adenoviral vector within the lung *via* the deep respiratory delivery mediated by endotracheal inoculation, we evaluated the relationship of Ad-vectored vaccine biodistribution to vaccine immunogenicity. To this end, mice were immunized with an adenoviral-vectored TB vaccine, AdHu5Ag85A, by either I.N. or I.T. method. Mice were sacrificed four weeks post-immunization to assess antigen (Ag)-specific T cell responses (**Figure 3A**). We first assessed airway mononuclear cells from the whole lung (both left and right lungs) obtained by the bronchoalveolar lavage (BAL), with a focus on examining antigen 85A-specific CD8 T cell responses *via* CD8 T cell tetramer-immunostaining (CD8^+^Tet^+^) or intracellular cytokine-staining (CD4^+^IFNγ, or CD8^+^IFNγ^+^) following *ex vivo* stimulation with Ag85A peptides (see gating strategy, **Supplementary Figure 1**). To define the gates and the background immunostaining, BAL cells from unimmunized mice were subjected to tetramer staining or BAL cells from immunized mice were cultured with control media (unstimulated) prior to IFNγ intracellular staining (see the top row of **Figure 3C**). Although deep respiratory delivery of the vaccine *via* I.T. inoculation induced a significant increase in total numbers of mononuclear cells in the airways compared to animals that received the vaccine *via* I.N inoculation (**Figure 3B**), comparable antigen-specific responses were induced in the BAL by either vaccine delivery method (**Figures 3C, D**).

**Figure 4:**
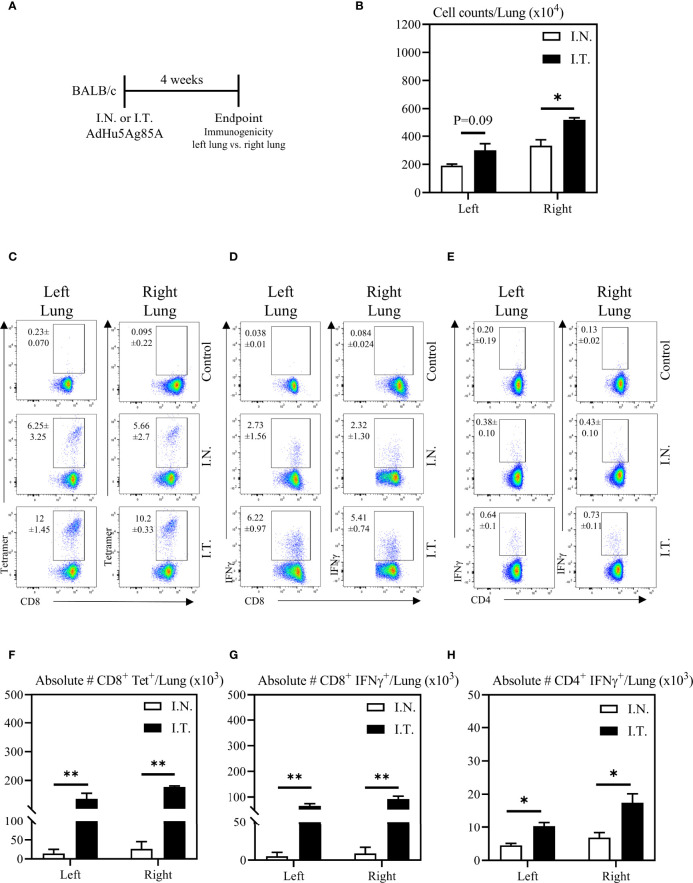
Vaccine-specific T cell responses in the left and right lung tissues following endotracheal immunization compared to intranasal immunization. Experimental schema **(A)**. Mice immunized intranasally (I.N.) or endotracheally (I.T.) with AdHu5Ag85A were scarified 4 weeks post-immunization and mononuclear cells from left and right lung homogenates were examined for vaccine-specific responses separately. **(B)** Bar graphs comparing total numbers of mononuclear cells in left and right lung tissues. **(C–E)** Representative flow cytometric dotplots showing frequencies of Ag85A-specific CD8 T cells (CD8+tet+) determined by tetramer staining, and frequencies of IFNγ+ CD8+ and CD4+ T cells determined by intracellular cytokine staining of cells stimulated with Ag85A CD8 or CD4 T-cell specific peptides in left and right lung tissues. Top row dotplots (Control) show the defining gates for tetramer population gated out of total CD8 T cells from unimmunized animal and the gates for CD8+IFNγ+ and CD4+IFNγ+ T cells out of total unstimulated CD8 and CD4 T cells from left and right lung tissues of immunized mice. Numericals indicated in the dotplots represent the mean frequency of parent (CD4 or CD8 T cells) ± SEM. **(F–H)** Bar graphs comparing absolute numbers of CD8+tet+, CD8+IFNγ+, and CD4+IFNγ+ T cells in left and right lung tissue of intranasal and endotracheal-immunized mice. Absolute numbers of CD8+tetramer+, CD8+ IFN-γ+ and CD4+ IFN-γ+ cells were calculated based on frequencies of CD3+live cells gated out of total events to exclude all non-immune cells. Data is from 3 mice/group, representative of two independent experiments and presented as mean ± SEM.*p < 0.05, **p < 0.01.

To further evaluate the relationship between vaccine biodistribution and vaccine immunogenicity, we next assessed immune responses independently in the left and right lung tissues. Mice were vaccinated as described above, and left and right lung tissues were isolated for immune analysis (**Figure 4A**). Appropriate controls were set up to define the gates and the background immunostaining as described for BAL cells (see the top row of **Figures 4C-E**). In addition, lung tissue sections were evaluated for histological changes following these two methods of vaccination. In agreement with increased vaccine biodistribution following I.T. vaccine delivery (**Figure 2**), we observed significantly greater total cell counts in the right lung following I.T. immunization with AdHu5Ag85A, compared to I.N. delivery (**Figure 4B**). Furthermore, there were both significantly increased frequencies and absolute numbers of antigen-specific tet+ CD8 T cells (**Figures 4C, F**) and IFNγ+ CD8 (**Figures 4D, G**) and CD4 (**Figures 4E, H**) both in the left and right lungs of I.T. immunized animals than in I.N. animals.

Additionally, the increased cellular infiltration in the lung of I.T. vaccine group was due to the vaccine and was not associated with the procedure itself since the comparable total lung cell counts ranging 3-5 millions/lung were seen between PBS I.T.-treated and untreated naïve mice. These data together suggest that the extent of vaccine biodistribution in the lung is positively correlated with enhanced vaccine-specific immune responses within respiratory mucosal tissue compartments, which was particularly evident in both the left and right lungs.

### Enhanced Protection Against Pulmonary Tuberculosis Following Endotracheal Immunization Compared to Intranasal Immunization

**Figure 5:**
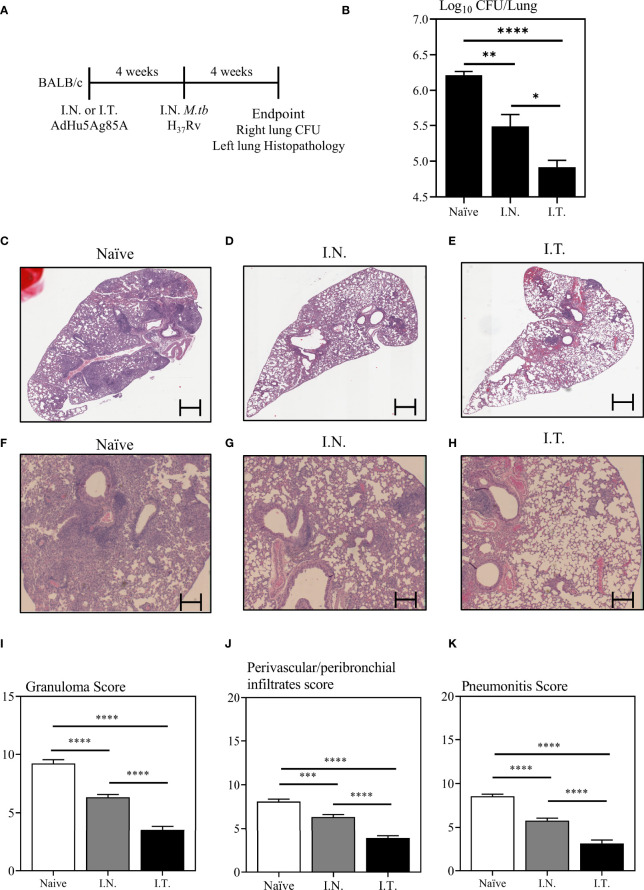
Immune protection against pulmonary tuberculosis following endotracheal immunization compared to intranasal immunization. Experimental schema **(A)**. Mice immunized intranasally (I.N.) or endotracheally (I.T.) with AdHu5Ag85A were infected with virulent *M. tuberculosis* (Mtb) and sacrificed 4 weeks post-infection. Unimmunized mice were included as controls (Naïve). Right lung homogenates were serially diluted and plated for the assessment of mycobacterial burden (colony forming unit -CFU). **(B)** Bar graph comparing Log_10_ CFU/lung in unimmunized (naïve), or I.N. or I.T. immunized mice. Data is from n = 6 mice/group and presented as mean ± SEM. *p < 0.05; **p <0.01; ****p < 0.0001. **(C–H)** Following *M.tb* infection, lungs were processed for H&E staining and examined for immunopathological changes. Representative low-power micrographs showing overall lung architectural changes and higher-power micrographs showing granuloma, areas of pneumonitis and peribronchial/perivascular infiltrates. **(I–K)** Bar graphs comparing the semi-quantitative scoring of the extent of lung granuloma, pneumonitis and infiltration of cells in naïve, I.N. and I.T. immunized mice. Scoring was carried out on a scale of 1 to 10 and independently verified by another researcher blinded to the experimental groups. Data is from n = 6 mice/group. Data in bar graphs are presented as mean ± SEM. ***p <0.001; ****p < 0.0001.

To examine whether improved vaccine-specific immunogenicity in the lung by broader respiratory biodistribution of AdHu5Ag85A would translate to enhanced protection against pulmonary TB, mice were immunized with AdHu5Ag85A by I.N. or I.T. delivery method (**Figure 5A**). A set of mice were left unimmunized as controls (Naïve). Four weeks after immunization, mice were challenged with virulent *M.tb*H37Rv. At four-week post-infection, mice were sacrificed. The right lung of each animal was harvested to assess mycobacterial burden by colony forming unit assay and the left lung was fixed in formalin for histopathological analysis after hematoxylin and eosin staining (**Figure 5A**). Since I.T. delivery led to significantly increased biodistribution of the vaccine primarily within the right lung over that by I.N delivery (**Figures 2B, C**), the assessment of *M.tb* bacterial burden in the right lung would more accurately address the relationship of increased vaccine biodistribution to the functional protective outcome of the vaccine. While both I.N. and I.T. immunization with AdHu5Ag85A significantly reduced the mycobacterial burden in the lung compared to the control (**Figure 5B**), I.T. immunization significantly enhanced protection as it further reduced the mycobacterial burden in the lung (~1.5 log reduction) over that by I.N. immunization (~0.8 log reduction) (**Figure 5B**).

To further examine vaccine-mediated protection, we assessed the lung immunopathology caused by *M.tb* infection. Indeed, in consistent with significantly reduced mycobacterial burden in the lung (**Figure 5B**), both I.N. and I.T. immunization markedly reduced lung histopathology (**Figures 5C-H**). Of importance, I.T. immunization further significantly reduced lung immunopathology over that by I.N. delivery both in microscopic changes (**Figures 5C-H**) and histological scoring of relative extent of granuloma formation, pneumonitis, and inflammatory infiltration (**Figures 5I-K**). The above data together indicate that endotracheal delivery of vaccine improves protection against pulmonary TB over intranasal delivery and such improved protection is associated with collectively enhanced biodistribution of vaccine within the lung.

## Discussion

Respiratory mucosal (RM) immunization has been regarded as a highly appealing route of vaccination against respiratory infections given its superiority in inducing protective mucosal immunity and its advantage of being a needle-free, and thereby pain-free approach (3). Currently, several novel vaccines such as viral-vectored vaccines for TB and COVID-19 are being clinically developed for respiratory mucosal delivery (6, 9–11, 30). However, pulmonary delivery methods in humans include both intranasal and inhaled aerosol methods. Although these methods are expected to result in differential deposition sites of vaccine within the respiratory tract and subsequently, differential immune responses and protection, it is difficult to directly investigate it in humans. As a result, our understanding of the relationship of vaccine biodistribution following different methods of respiratory delivery to vaccine immunogenicity and protection has remained to be limited. In the current study, using an adenovirus-vectored TB vaccine (AdHu5Ag85A) as a model vaccine, we find that endotracheal delivery is superior to intranasal delivery in rendering wide and deep lung biodistribution of vaccine and subsequently, enhanced vaccine-specific T cell responses and protective efficacy against pulmonary *M.tb* challenge.

Clinically, it was found that only inhaled aerosol, but not parenteral, vaccination induced significant respiratory mucosal immunity (9, 10). Since the inhaled aerosol technology bypasses the nasal passage, and generates and directly deposits mostly 2-5 µm size aerosol vaccine particles deep into human respiratory tract (airways) (10), these clinical observations suggest the immune efficacy by this respiratory mucosal delivery method in humans. Here for the first time, our experimental data reveals a causal relationship of relative depth and width of airway vaccine deposition to vaccine immunogenicity and protective efficacy following intranasal vs. endotracheal delivery. Our observations thus provide a potential explanation for unsatisfactory efficacy observed in human adults following intranasal delivery of flu vaccine (5, 6) and support the advantage of inhaled aerosol delivery method over intranasal delivery for human applications.

Other two previous studies that compared intranasal and intratracheal vaccine delivery did not assess vaccine biodistribution (17, 18). For instance, Minne et al. employed ovalbumin (OVA) as a surrogate and studied the influenza vaccine deposition regions in the lung by measuring OVA content in the nasal washes and lung homogenates (18). However, given that the cell tropism of a viral-vectored vaccine plays a critical role, using a surrogate that resembles the vaccine is important when evaluating biodistribution. In this regard, in our study, using a surrogate of the same viral vector as that used in the vaccine but expressing luciferase, we reliably demonstrated differential biodistribution following different methods of pulmonary delivery. Although De Swart et al. used the measles virus vaccine expressing enhanced green fluorescent protein to study the vaccine distribution, they only evaluated the bronchoalveolar cells and lung sections as a measure of vaccine distribution (17). As such they were unable to demonstrate the biodistribution.

It has now been fully established that vaccine induced CD4 and CD8 T cells residing at the respiratory mucosa plays a critical role in pulmonary immunity to pathogens by immediately encaging the pathogens at the site of infection (2, 3). Previous studies that assessed the impact of pulmonary delivery methods of vaccines on vaccine-induced immunity has shown that the delivery of vaccine to lower respiratory tract results in induction of higher titers of neutralizing antibodies (17, 18). Such knowledge cannot be generalized to induction of T cell immunity at the respiratory mucosa since the T cell responses in the lung are highly regulated to preserve the vital role of the lung, oxygen exchange. Here we show that endotracheal delivery of AdHu5Ag85A in animals yields heightened Ag-specific tetramer+ CD8+ T cells responses, IFN-γ production by CD4 and CD8 T cells in the lung and leads to enhanced protective efficacy against *M.tb* infection compared to intranasal immunization. Not only the reduction in bacterial load, but also markedly reduced histopathological damage in endotracheally immunized mice indicates that the deeper and wider biodistribution of vaccine is critical. Of note, although endotracheal delivery led to significantly greater biodistribution only in the right lung, it resulted in significantly increased antigen-specific T cell responses in both left and right lungs over those by intranasal immunization. These findings suggest that upon antigen-specific T cell priming in the local draining lymphoid tissues, similar numbers of T cells were recruited into the left and right lungs in response to the local inflammatory signals following endotracheal immunization.

Pulmonary delivery method of a vaccine for human application relies on various factors, such as vaccine formulation, *in vivo* tropism of the vaccine, and whether to target the upper or lower respiratory tract (3, 7, 8, 17). This is also reliant on the tropism of the pathogen against which the vaccine is directed to, and other parameters including duration of exposure to vaccine when delivered *via* inhalation or aerosolization, and the delivery device. In conclusion, we show that biodistribution of an adenoviral-vectored vaccine in the lung is dependent on pulmonary vaccine delivery method and that direct delivery of vaccine to deep into the respiratory tract of both lungs by endotracheal method increase the biodistribution and induce enhanced respiratory mucosal T cell immunity compared to intranasal delivery. Our study thus supports inhaled aerosol delivery over intranasal method for developing respiratory mucosal vaccine strategies for human application.

## Data Availability Statement

The raw data supporting the conclusions of this article will be made available by the authors, without undue reservation.

## Ethics Statement

The animal study was reviewed and approved by The Animal Research and Ethics Board at McMaster University.

## Author Contributions

VJ, SA, MD’A, AZ, XF, MJ performed experiments or provided technical assistance. MT, ZX conceived the project. VJ, SA, MJ performed data analysis. VJ, MJ, ZX wrote the manuscript. All authors contributed to the article and approved the submitted version.

## Funding

This study was supported by funds from The Canadian Institutes for Health Research and the Natural Sciences and Engineering Research Council of Canada (FDN-154316).

## Conflict of Interest

The authors declare that the research was conducted in the absence of any commercial or financial relationships that could be construed as a potential conflict of interest.

## Publisher’s Note

All claims expressed in this article are solely those of the authors and do not necessarily represent those of their affiliated organizations, or those of the publisher, the editors and the reviewers. Any product that may be evaluated in this article, or claim that may be made by its manufacturer, is not guaranteed or endorsed by the publisher.

## Supplementary Material

Supplementary Figure 1 Dotplots depicting gating strategy used for flowcytometry data analysis of immune responses in the airways and lung tissue. Live CD3+ cells were gated after removing doublets. Debris were then gated out from Live CD3+ cells before identifying CD4+ and CD8+ T-cells for downstream gating of tetramer and IFNγ+ populations.
