# Corrigendum: Differential biodistribution of adenoviral-vectored vaccine following intranasal and endotracheal deliveries leads to different immune outcomes

**DOI:** 10.3389/fimmu.2023.1151809

**Published:** 2023-02-07

**Authors:** Vidthiya Jeyanathan, Sam Afkhami, Michael R. D’Agostino, Anna Zganiacz, Xueya Feng, Matthew S. Miller, Mangalakumari Jeyanathan, Michael R. Thompson, Zhou Xing

**Affiliations:** ^1^ McMaster Immunology Research Centre, M. G. DeGroote Institute for Infectious Disease Research, Hamilton, ON, Canada; ^2^ Department of Medicine, McMaster University, Hamilton, ON, Canada; ^3^ Department of Biochemistry & Biomedical Sciences, McMaster University, Hamilton, ON, Canada; ^4^ Department of Chemical Engineering, McMaster University, Hamilton, ON, Canada

**Keywords:** respiratory mucosal immunization, intranasal, endotracheal, biodistribution, Adenovirus-vectored vaccine, Tuberculosis, mucosal immunity, T cells

In the published article, an author name was incorrectly written as “Vidthiya Jeyananthan”. The correct spelling is “Vidthiya Jeyanathan”.

The authors apologize for this error and state that this does not change the scientific conclusions of the article in any way. The original article has been updated.

