# Supplementary figures and images for: Differential Biodistribution of Adenoviral-Vectored Vaccine Following Intranasal and Endotracheal Deliveries Leads to Different Immune Outcomes
Source: Front Immunol. 2022 Jun 10;13:860399. doi: 10.3389/fimmu.2022.860399 (PMC9231681; doi:10.3389/fimmu.2022.860399)

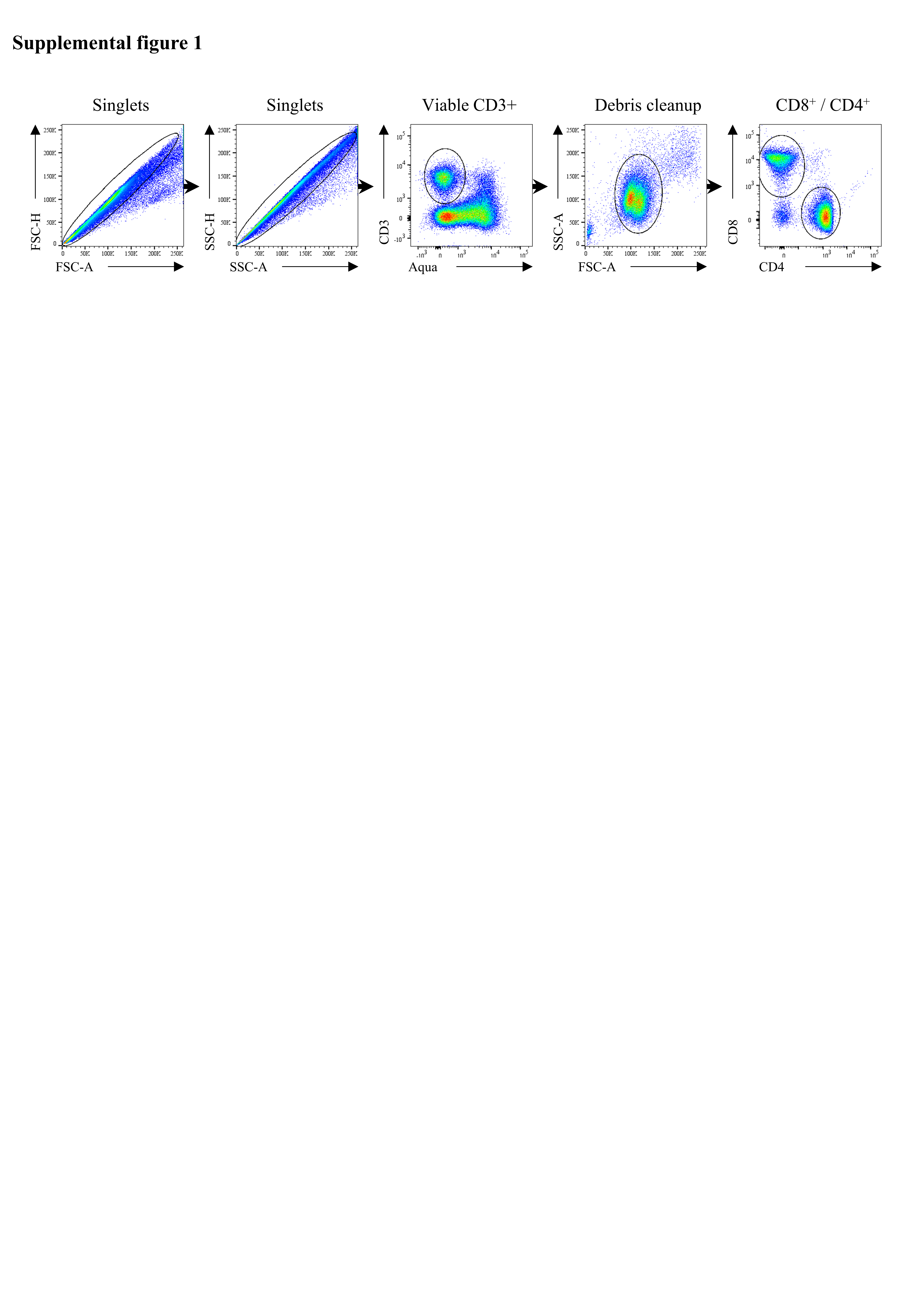

Supplement: Supplementary Figure 1 — Dotplots depicting gating strategy used for flowcytometry data analysis of immune responses in the airways and lung tissue. Live CD3+ cells were gated after removing doublets. Debris were then gated out from Live CD3+ cells before identifying CD4+ and CD8+ T-cells for downstream gating of tetramer and IFNγ+ populations. [file Image_1.tif]
